# Bis[tris­(phenanthroline-κ^2^ *N*,*N*′)cobalt(II)] undeca­tungsto(VI)vanado(V)phosphate dihydrate

**DOI:** 10.1107/S160053681400484X

**Published:** 2014-03-08

**Authors:** Assia Hajsalem, Sameh Aoun, Aurelien Planchat, Mohamed Rzaigui, Samah Akriche Toumi

**Affiliations:** a Laboratoire de Chimie des Matériaux, Faculté des Sciences de Bizerte, 7021 Zarzouna Bizerte, Tunisia; b Laboratoire CEISAM, UMR CNRS 6230, UFR des Sciences et des Techniques, 2 rue de la Houssinière, BP 92208, 44322 Nantes Cedex 03, France

## Abstract

In the title hydrated salt, [Co(C_12_H_8_N_2_)_3_]_2_[PVW_11_O_40_]·2H_2_O, the complete Kegggin ion is generated by crystallographic inversion symmetry, which imposes statistical disorder on the O atoms of its central PO_4_ group. The V atom is statistically disordered over all the metal sites of the anion. In the cation, the Co^2+^ ion is coordinated by three bidentate 1,10-phenanthroline (phen) ligands, generating a distorted CoN_6_ octa­hedron. Possible very weak intra­molecular C—H⋯π inter­actions occur in the cation. In the crystal, the components are linked by O—H⋯O and C—H⋯O inter­actions, building a three-dimensional network featuring one-dimensional voids along the *c*-axis direction.

## Related literature   

For related vanadium-substituted Keggin-ion structures, see: Glinskaya *et al.* (1989 ▶); Klevtsova *et al.* (1990 ▶, 1991 ▶); Li *et al.* (2008 ▶); Radkov & Beer (1995 ▶). For IR spectroscopy investigations of Keggin ions, see: Lee & Misono (1997 ▶); Deltcheff *et al.* (1983 ▶); Watras & Teplyakov (2005 ▶). For bond-valence calculations, see: Brown & Altermatt (1985 ▶). For background to polyoxidometalate chemistry, see: Pope & Müller (1991 ▶, 1994 ▶).
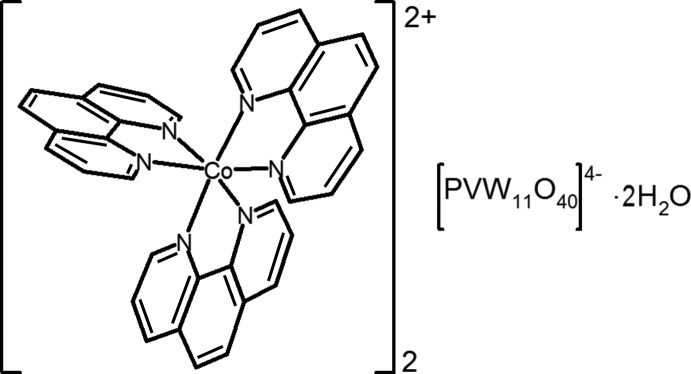


## Experimental   

### 

#### Crystal data  

[Co(C_12_H_8_N_2_)_3_][PVW_11_O_40_]·2H_2_O
*M* *_r_* = 3979.38
Monoclinic, 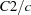

*a* = 19.487 (2) Å
*b* = 18.049 (3) Å
*c* = 25.216 (2) Å
β = 100.22 (3)°
*V* = 8728.4 (18) Å^3^
*Z* = 4
Mo *K*α radiation
μ = 15.02 mm^−1^
*T* = 295 K
0.13 × 0.08 × 0.04 mm

#### Data collection  

Nonius KappaCCD diffractometer
Absorption correction: multi-scan (*SORTAV*; Blessing, 1995 ▶) *T*_min_ = 0.202, *T*_max_ = 0.421
62427 measured reflections
11235 independent reflections
8172 reflections with *I* > 2σ(*I*)
*R*_int_ = 0.070

#### Refinement  

*R*[*F* ^2^ > 2σ(*F* ^2^)] = 0.054
*wR*(*F* ^2^) = 0.095
*S* = 1.16
11235 reflections
688 parameters
3 restraints
H atoms treated by a mixture of independent and constrained refinement
Δρ_max_ = 1.39 e Å^−3^
Δρ_min_ = −1.51 e Å^−3^

### 

Data collection: *COLLECT* (Hooft, 1998 ▶); cell refinement: *DIRAX/LSQ* (Duisenberg *et al.*, 2000 ▶); data reduction: *EVALCCD* (Duisenberg *et al.*, 2003 ▶); program(s) used to solve structure: *SHELXS97* (Sheldrick, 2008 ▶); program(s) used to refine structure: *SHELXL97* (Sheldrick, 2008 ▶); molecular graphics: *ORTEP-3 for Windows* (Farrugia, 2012 ▶) and *DIAMOND* (Brandenburg & Putz, 2005 ▶); software used to prepare material for publication: *WinGX* (Farrugia, 2012 ▶).

## Supplementary Material

Crystal structure: contains datablock(s) I. DOI: 10.1107/S160053681400484X/hb7203sup1.cif

Structure factors: contains datablock(s) I. DOI: 10.1107/S160053681400484X/hb7203Isup2.hkl

CCDC reference: 989480

Additional supporting information:  crystallographic information; 3D view; checkCIF report

## Figures and Tables

**Table 1:** Selected bond lengths (Å)

Co—N3	2.053 (9)
Co—N6	2.060 (8)
Co—N1	2.066 (10)
Co—N4	2.066 (11)
Co—N2	2.078 (10)
Co—N5	2.085 (8)

**Table 2:** Hydrogen-bond geometry (Å, °) *Cg*1 and *Cg*2 are the centroids of the N1/C1–C4/C12 and N3/C13–C16/C24 rings, respectively.

*D*—H⋯*A*	*D*—H	H⋯*A*	*D*⋯*A*	*D*—H⋯*A*
O1*W*—H1*W*1⋯O1*E*	0.85 (1)	2.17 (11)	2.928 (14)	149 (20)
O1*W*—H2*W*1⋯O3*E* ^i^	0.85 (1)	1.99 (3)	2.836 (13)	173 (17)
C9—H9⋯O5*E* ^ii^	0.93	2.55	3.208 (15)	128
C26—H26⋯O12^iii^	0.93	2.46	2.973 (15)	114
C33—H33⋯O5^iv^	0.93	2.53	3.345 (13)	147
C34—H34⋯O8	0.93	2.43	3.114 (13)	130
C34—H34⋯*Cg*1	0.93	3.04	3.811 (12)	142
C25—H25⋯*Cg*2	0.93	2.99	3.777 (13)	143

Symmetry codes: (i) 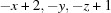; (ii) 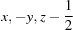; (iii) 
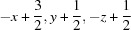
; (iv) 
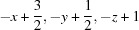
.

**Fig. 1.**
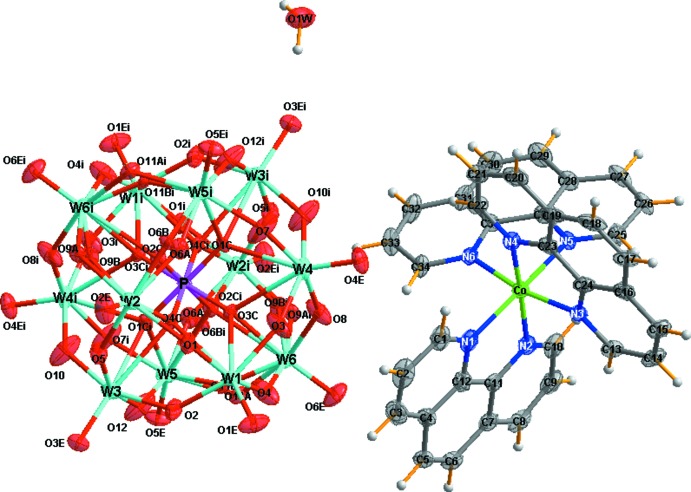
An ORTEP view of (I) with displacement ellipsoids drawn at the 30% probability level. H atoms are represented as small spheres of arbitrary radii. Hydrogen bonds are represented as dashed lines. [Symmetry code: (i) 1 - x, 1 - y, 1 - z]

**Fig. 2.**
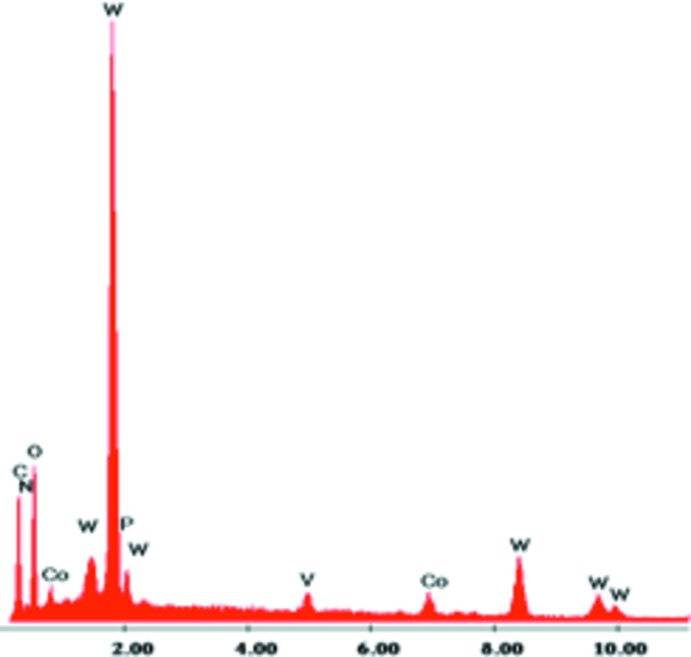
EDAX pattern of I

**Fig. 3.**
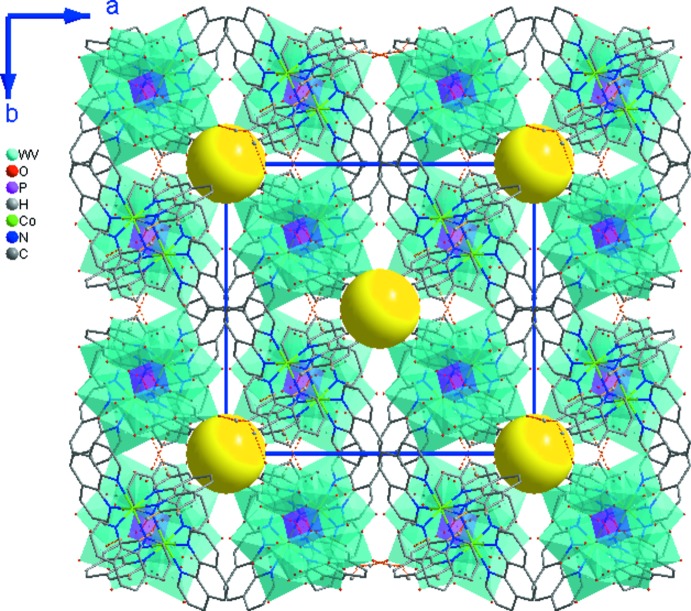
Packing diagram of I viewed along c axis showing a three-dimensional-supramolecular structure featuring the voids represented as large yellow ball. The H-atoms not included in H-bond scheme are omitted.

**Fig. 4.**
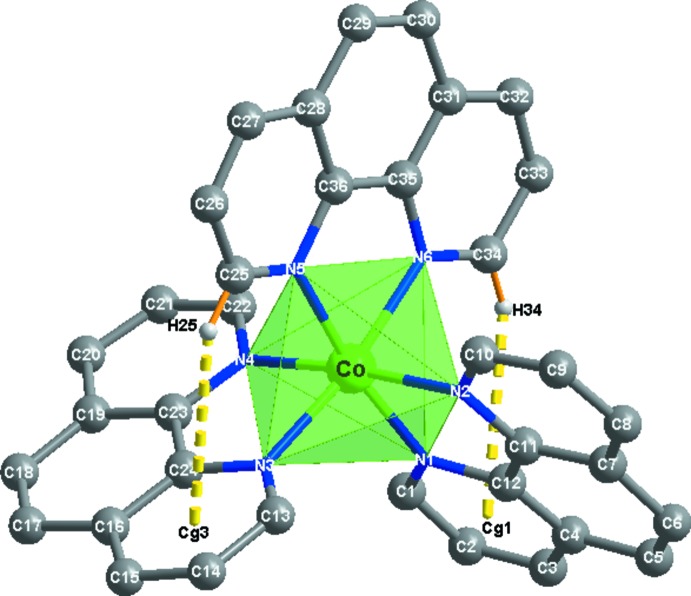
View of intramolecular C—H···π interaction in (I). The H-atoms not included in H-bond scheme are omitted.

## supplementary crystallographic information

### 1. Comment

A bibliographic survey shows only a few examples of substituted vanadium keggin-type [XV_1_W_11_O_40_]*^p^*^-^ clusters associated to organometallic or organic moieties: such as (C_2_N_2_H_10_)_2_[VV_1_W_11_O_40_]·6H_2_O (Glinskaya *et al.*, 1989), [N(CH_3_)_4_]_4_[VV_1_W_11_O_40_]·4.5H_2_O (Klevtsova *et al.*, 1991),[(CH_3_)_2_NCHO]_4_H_4_[VV_1_W_11_O_40_].2[(CH_3_)_2_NCHO]·2H_2_O (Klevtsova *et al.*, 1990), [Cu(phen)_2_]_2_[PVW_11_O_40_] (phen = phenanthroline)(Li *et al.*, 2008),(n-Bu_4_N)_4_[PMW_11_O_40_] (n-Bu = *n*-Butyl and *M* = V, Nb, Ta) (Radkov & Beer, 1995). Here, we report a new monosubstituted vanadium tungstophosphate Keggin-type cluster decorated by mononuclear metal-organic complex [Co(phen)_3_]_2_[PVW_11_O_40_]·2H_2_O (phen = phenanthroline) (I).

The asymmetric unit of of I consists of a mononuclear complex [Co(phen)_3_]^2+^ cation and a half of Keggin-type [PVW_11_O_40_]^4-^ anion and one water molecule. As P atom is located on centre of inversion symmetry, the whole [PVW_11_O_40_]^4-^ polyoxidoanion is generated by this element and so is composed of a disordered PO_4_ tetrahedron surrounded by four vertex-sharing *M*_3_O_13_ (with *M* = W/V) subunits which result from the association of three edge-sharing MO_6_ octahedra enwrapping a PO_8_ cube with oxygen atom site occupancy of 0.5. In the MO_6_ (with *M* = W/V) octahedra, the position of metal atom is crystallographically disordered and constrained as 11/12 W and 1/12 V, with occupancies of 0.083 and 0.917 for W and V respectively.

The contents of W and V revealed by X-ray analysis are consistent with the results from elemental analyses and scanning electronic microscopy (Fig. 2) as well as the IR spectroscopy wich shows that the streching vibration of (P—O) splits into two absoption bands at 1095 cm^-1^ and 1068 cm^-1^ because of the lower symmetry so as well confirm the presence of monosubstituted vanadium keggin-type clusters in I (Lee *et al.*, 1997; Deltcheff *et al.*, 1983; Watras *et al.*, 2005).

The assignment of oxidation states for the tungsten and vanadium atoms is confirmed by bond valence sum calculations (Brown & Altermatt, 1985)) which show that vanadium atom has +V oxidation state (average 4.98 valence units) while tungsten atoms have +VI oxidation state (average 6.23 valence units). These oxidation states are identical with the charge balance considerations and so consistent with the expected [PV^+V^W^+VI^_11_O_40_]^4-^ subunits.

The P—O bond distances range 1.481 (11)—1.582 (11) Å and O—P—O bond angles interval 105.5 (6)—112.6 (6) °. Commonly, the M—O bond distances are grouped into three sets: M—Ot, M—Ob and M—Oc (with Ot: terminal oxygen atoms, Oc: central oxygen atoms, Ob: bridging oxygen atoms) which are respectively ranged between 1.667 (8)—1.677 (7) Å, 2.396 (11)—2.526 (12) Å and 1.750 (18)—2.085 (14) Å. With regard to the mononuclear complex,the Co^2+^ metal is also coordinated by six nitrogen atoms from three chelating 1,10-phenanthroline ligands to form a slightly distorted MN_6_ octahedron with bond lenghts around Co, are 2.053 (9)—2.085 (8) Å (Co—N) and 80.0 (4)—173.9 (4)° (N—Co—N) (Table 1).

The crystal packing of I shows that the discrete polyoxidoanion subunits are interconnected through water molecules *via* O—H···O hydrogen bonding interactions with O···O separation ranging from 2.836 (13) to 2.928 (14) Å (Table 2), to perform alternating [PVW_11_O_40_(H_2_O)_2_]^4n-^ ribbons extending along [110] and [110] crystallographic directions. The so-obtained one-dimensional-subnetworks stack together by the metal-organic moieties thanks to weak C–H···O (mean C···O = 3.144 Å) (Table 1) and electrostatic interactions so as to build three-dimensional-supramolecular network generating vacant one-dimensional-channels along *c* axis as can be seen in Fig. 3. Very weak intramolecular C—H···π interactions of phen rings (Fig. 4) with mean distances of 3 Å (Table 2) are also observed.

### 2. Experimental

A reaction mixture of Na_2_WO_4_·2H_2_O (2 g, 6.064 mmol), NaH_2_PO_4_·2H_2_O (0.1034 g, 0.6628 mmol), CoCl_2_·6H_2_O (0.1951 g, 0.8196 mmol), V_2_O_5_ (0,0455 g; 0.25 mmol) and phen·H_2_O (0.3196 g, 1.7758 mmol) were added to water (10 ml). The mixture was adjusted to pH = 5.5 by the addition of 4*M* HCl aqueous solution then stirred for 30 min in air. The mixture solution was transferred into a 23 ml Teflon-lined autoclave and crystallized at 180°C for 4 days. Then the autoclave was cooled at 10°C.h^-1^ to room temperature. The resulting dark yellow block crystals of I were filtered off, washed with water, and dried at ambient temperature to give yields of 68% based on W. Anal. Calc. For C_72_H_52_N_12_Co_2_O_42_PVW_11_ (%): C 21.79, H 1.27, N 4.20, W 50.92, V 1.28, P 0.78, Co 2.96; Found C 21.71, H 1.31, N 4.25, W 50.80, V 1.31, P 3/4, Co 2.98; IR (KBr, cm^-1^): 966 ν(*M*=Ot), 887 ν(M—Ob—*M*), 799 ν(M—Oc—*M*) with *M*=W/V and 1095 and 1068 ν(P—O).

### 3. Refinement

All H atoms attached to C atoms were fixed geometrically and treated as riding, with C—H = 0.93 Å and *U*_iso_(H) = 1.2Ueq(C). Water H atoms were refined using restraints [O—H = 0.85 (1) A °, H···H = 1.44 (2) A ° and *U*_iso_(H) = 1.5Ueq(O)].

Many trials of crystal growing are unsuccessful and despite the good quality of selected crystal for experimental X-Ray,the largest isolated one has a relatively small size (crystal size: 0.04 × 0.08 × 0.013 mm), which lead to the poor diffraction at higher angles.

### Crystal data

**Table:**

[Co(C_12_H_8_N_2_)_3_][PVW_11_O_40_]·2H_2_O	*F*(000) = 7240
*M**_r_* = 3979.38	*D*_x_ = 3.028 Mg m^−^^3^
Monoclinic, *C*2/*c*	Mo *K*α radiation, λ = 0.71073 Å
*a* = 19.487 (2) Å	Cell parameters from 25 reflections
*b* = 18.049 (3) Å	θ = 9–11°
*c* = 25.216 (2) Å	µ = 15.02 mm^−^^1^
β = 100.22 (3)°	*T* = 295 K
*V* = 8728.4 (18) Å^3^	PRISM, yellow
*Z* = 4	0.13 × 0.08 × 0.04 mm

### Data collection

**Table:**

Nonius KappaCCD diffractometer	11235 independent reflections
Radiation source: fine-focus sealed tube	8172 reflections with *I* > 2σ(*I*)
Horizonally mounted graphite crystal monochromator	*R*_int_ = 0.070
Detector resolution: 9 pixels mm^-1^	θ_max_ = 28.8°, θ_min_ = 6.4°
CCD rotation images, thick slices scans	*h* = −26→25
Absorption correction: multi-scan (*SORTAV*; Blessing, 1995)	*k* = −24→24
*T*_min_ = 0.202, *T*_max_ = 0.421	*l* = −34→32
62427 measured reflections	

### Refinement

**Table:**

Refinement on *F*^2^	Primary atom site location: structure-invariant direct methods
Least-squares matrix: full	Secondary atom site location: difference Fourier map
*R*[*F*^2^ > 2σ(*F*^2^)] = 0.054	Hydrogen site location: inferred from neighbouring sites
*wR*(*F*^2^) = 0.095	H atoms treated by a mixture of independent and constrained refinement
*S* = 1.16	*w* = 1/[σ^2^(*F*_o_^2^) + 250.0304*P*] where *P* = (*F*_o_^2^ + 2*F*_c_^2^)/3
11235 reflections	(Δ/σ)_max_ = 0.002
688 parameters	Δρ_max_ = 1.39 e Å^−^^3^
3 restraints	Δρ_min_ = −1.51 e Å^−^^3^

### Special details

**Table:**

Geometry. All e.s.d.'s (except the e.s.d. in the dihedral angle between two l.s. planes) are estimated using the full covariance matrix. The cell e.s.d.'s are taken into account individually in the estimation of e.s.d.'s in distances, angles and torsion angles; correlations between e.s.d.'s in cell parameters are only used when they are defined by crystal symmetry. An approximate (isotropic) treatment of cell e.s.d.'s is used for estimating e.s.d.'s involving l.s. planes.
Refinement. Refinement of *F*^2^ against ALL reflections. The weighted *R*-factor *wR* and goodness of fit *S* are based on *F*^2^, conventional *R*-factors *R* are based on *F*, with *F* set to zero for negative *F*^2^. The threshold expression of *F*^2^ > σ(*F*^2^) is used only for calculating *R*-factors(gt) *etc*. and is not relevant to the choice of reflections for refinement. *R*-factors based on *F*^2^ are statistically about twice as large as those based on *F*, and *R*- factors based on ALL data will be even larger.

### Fractional atomic coordinates and isotropic or equivalent isotropic displacement parameters (Å^2^)

**Table:**

	*x*	*y*	*z*	*U*_iso_*/*U*_eq_	Occ. (<1)
W1	0.90431 (2)	0.16939 (2)	0.468951 (18)	0.03506 (10)	0.91
V1	0.90431 (2)	0.16939 (2)	0.468951 (18)	0.03506 (10)	0.09
W2	0.91248 (2)	0.27877 (3)	0.587700 (17)	0.03574 (11)	0.91
V2	0.91248 (2)	0.27877 (3)	0.587700 (17)	0.03574 (11)	0.09
W3	0.83223 (2)	0.10207 (3)	0.579002 (19)	0.04092 (12)	0.92
V3	0.83223 (2)	0.10207 (3)	0.579002 (19)	0.04092 (12)	0.08
W4	0.82438 (2)	0.32068 (3)	0.391898 (18)	0.04283 (12)	0.92
V4	0.82438 (2)	0.32068 (3)	0.391898 (18)	0.04283 (12)	0.08
W5	0.67180 (3)	0.07254 (3)	0.48920 (2)	0.04756 (13)	0.92
V5	0.67180 (3)	0.07254 (3)	0.48920 (2)	0.04756 (13)	0.08
W6	0.74373 (3)	0.14331 (3)	0.380723 (19)	0.04799 (13)	0.92
V6	0.74373 (3)	0.14331 (3)	0.380723 (19)	0.04799 (13)	0.08
O1E	0.9773 (4)	0.1310 (5)	0.4547 (4)	0.072 (3)	
O2E	0.9883 (4)	0.2925 (5)	0.6293 (3)	0.064 (2)	
O3E	0.8718 (5)	0.0319 (5)	0.6152 (3)	0.066 (2)	
O4E	0.8576 (4)	0.3557 (5)	0.3403 (3)	0.057 (2)	
O5E	0.6360 (4)	−0.0117 (4)	0.4862 (3)	0.052 (2)	
O6E	0.7424 (5)	0.0939 (5)	0.3244 (3)	0.069 (3)	
O1	0.9412 (4)	0.2330 (4)	0.5277 (3)	0.051 (2)	
O2	0.8823 (4)	0.1027 (4)	0.5215 (3)	0.053 (2)	
O3	0.8945 (5)	0.2558 (5)	0.4245 (3)	0.073 (3)	
O4	0.8351 (5)	0.1247 (5)	0.4175 (4)	0.079 (3)	
O5	0.8869 (4)	0.1821 (4)	0.6090 (3)	0.053 (2)	
O6A	0.8877 (8)	0.3591 (10)	0.5382 (7)	0.038 (4)	0.50
O6B	0.9132 (8)	0.3772 (9)	0.5575 (6)	0.030 (3)	0.50
O7	0.8556 (4)	0.3900 (6)	0.4469 (4)	0.081 (3)	
O8	0.7761 (5)	0.2355 (5)	0.3599 (3)	0.072 (3)	
O9A	0.8604 (7)	0.3245 (9)	0.6394 (6)	0.032 (3)	0.50
O9B	0.8340 (7)	0.3084 (10)	0.6198 (6)	0.035 (4)	0.50
O10	0.7634 (4)	0.1309 (7)	0.6194 (4)	0.086 (4)	
O11A	0.7035 (10)	0.0633 (11)	0.4178 (7)	0.041 (4)	0.50
O11B	0.7226 (10)	0.0845 (10)	0.4384 (7)	0.038 (4)	0.50
O12	0.7599 (4)	0.0525 (6)	0.5323 (4)	0.081 (3)	
P	0.7500	0.2500	0.5000	0.0229 (6)	
O1C	0.7595 (6)	0.3206 (7)	0.4667 (4)	0.028 (3)	0.50
O3C	0.7921 (6)	0.2294 (7)	0.4564 (5)	0.028 (3)	0.50
O4C	0.8176 (6)	0.2148 (7)	0.5224 (4)	0.027 (3)	0.50
O2C	0.7968 (6)	0.3041 (7)	0.5406 (4)	0.028 (3)	0.50
O1W	1.0714 (7)	0.1131 (6)	0.3771 (6)	0.098 (4)	
H1W1	1.033 (6)	0.115 (9)	0.388 (8)	0.147*	
H2W1	1.092 (8)	0.071 (5)	0.380 (9)	0.147*	
Co	0.80233 (7)	0.31178 (8)	0.15603 (5)	0.0367 (3)	
N1	0.8600 (5)	0.2468 (6)	0.2151 (4)	0.051 (2)	
N2	0.7596 (5)	0.2088 (5)	0.1326 (4)	0.047 (2)	
N3	0.8759 (5)	0.3135 (5)	0.1070 (3)	0.041 (2)	
N4	0.8545 (6)	0.4082 (6)	0.1827 (4)	0.054 (3)	
N5	0.7316 (4)	0.3680 (5)	0.0981 (3)	0.0375 (19)	
N6	0.7208 (4)	0.3243 (5)	0.1973 (3)	0.0365 (19)	
C1	0.9090 (6)	0.2668 (9)	0.2556 (5)	0.064 (4)	
H1	0.9240	0.3158	0.2573	0.077*	
C2	0.9398 (8)	0.2178 (13)	0.2961 (6)	0.083 (5)	
H2	0.9743	0.2340	0.3239	0.099*	
C3	0.9184 (9)	0.1468 (12)	0.2937 (6)	0.085 (5)	
H3	0.9379	0.1135	0.3203	0.102*	
C4	0.8660 (7)	0.1226 (8)	0.2505 (5)	0.061 (3)	
C5	0.8418 (9)	0.0467 (8)	0.2431 (6)	0.075 (4)	
H5	0.8620	0.0104	0.2671	0.089*	
C6	0.7912 (9)	0.0282 (8)	0.2025 (6)	0.073 (4)	
H6	0.7755	−0.0205	0.1995	0.088*	
C7	0.7601 (7)	0.0823 (7)	0.1630 (5)	0.060 (3)	
C8	0.7059 (8)	0.0667 (8)	0.1192 (6)	0.070 (4)	
H8	0.6881	0.0190	0.1136	0.084*	
C9	0.6808 (8)	0.1224 (9)	0.0859 (6)	0.072 (4)	
H9	0.6444	0.1135	0.0574	0.086*	
C10	0.7080 (7)	0.1908 (8)	0.0936 (5)	0.060 (3)	
H10	0.6891	0.2279	0.0698	0.072*	
C11	0.7863 (6)	0.1554 (6)	0.1681 (4)	0.046 (3)	
C12	0.8393 (6)	0.1741 (8)	0.2123 (5)	0.054 (3)	
C13	0.8843 (6)	0.2656 (7)	0.0680 (5)	0.052 (3)	
H13	0.8576	0.2226	0.0639	0.062*	
C14	0.9317 (6)	0.2776 (8)	0.0331 (5)	0.059 (3)	
H14	0.9351	0.2440	0.0057	0.071*	
C15	0.9731 (6)	0.3399 (7)	0.0398 (5)	0.058 (3)	
H15	1.0055	0.3478	0.0174	0.069*	
C16	0.9669 (6)	0.3917 (7)	0.0805 (5)	0.051 (3)	
C17	1.0060 (6)	0.4588 (7)	0.0903 (6)	0.060 (3)	
H17	1.0397	0.4700	0.0696	0.072*	
C18	0.9948 (6)	0.5058 (7)	0.1291 (6)	0.064 (4)	
H18	1.0210	0.5491	0.1347	0.077*	
C19	0.9437 (6)	0.4915 (7)	0.1621 (5)	0.057 (3)	
C20	0.9284 (8)	0.5398 (8)	0.2027 (6)	0.066 (4)	
H20	0.9512	0.5851	0.2089	0.079*	
C21	0.8798 (9)	0.5191 (8)	0.2324 (6)	0.073 (4)	
H21	0.8715	0.5488	0.2607	0.087*	
C22	0.8431 (8)	0.4549 (7)	0.2207 (5)	0.063 (4)	
H22	0.8084	0.4435	0.2403	0.076*	
C23	0.9041 (6)	0.4266 (6)	0.1535 (4)	0.046 (3)	
C24	0.9158 (5)	0.3752 (6)	0.1129 (4)	0.043 (2)	
C25	0.7358 (7)	0.3868 (7)	0.0480 (5)	0.057 (3)	
H25	0.7775	0.3777	0.0361	0.068*	
C26	0.6827 (7)	0.4185 (7)	0.0127 (5)	0.058 (3)	
H26	0.6883	0.4298	−0.0222	0.069*	
C27	0.6220 (7)	0.4333 (7)	0.0292 (5)	0.059 (3)	
H27	0.5860	0.4561	0.0058	0.071*	
C28	0.6129 (6)	0.4147 (6)	0.0813 (4)	0.044 (3)	
C29	0.5491 (6)	0.4251 (8)	0.1015 (5)	0.063 (4)	
H29	0.5109	0.4472	0.0800	0.076*	
C30	0.5453 (6)	0.4023 (9)	0.1519 (6)	0.069 (4)	
H30	0.5036	0.4096	0.1643	0.083*	
C31	0.6020 (5)	0.3676 (8)	0.1873 (5)	0.054 (3)	
C32	0.6007 (6)	0.3421 (9)	0.2395 (5)	0.067 (4)	
H32	0.5605	0.3479	0.2541	0.080*	
C33	0.6570 (6)	0.3094 (8)	0.2688 (5)	0.060 (3)	
H33	0.6559	0.2934	0.3037	0.072*	
C34	0.7170 (6)	0.2996 (7)	0.2464 (4)	0.049 (3)	
H34	0.7552	0.2753	0.2662	0.058*	
C35	0.6646 (5)	0.3573 (6)	0.1679 (4)	0.036 (2)	
C36	0.6705 (5)	0.3812 (6)	0.1147 (4)	0.036 (2)	

### Atomic displacement parameters (Å^2^)

**Table:**

	*U*^11^	*U*^22^	*U*^33^	*U*^12^	*U*^13^	*U*^23^
W1	0.0286 (2)	0.0352 (2)	0.0414 (2)	0.00378 (18)	0.00621 (17)	−0.00271 (19)
V1	0.0286 (2)	0.0352 (2)	0.0414 (2)	0.00378 (18)	0.00621 (17)	−0.00271 (19)
W2	0.0295 (2)	0.0391 (2)	0.0366 (2)	0.00004 (18)	0.00030 (17)	0.00294 (18)
V2	0.0295 (2)	0.0391 (2)	0.0366 (2)	0.00004 (18)	0.00030 (17)	0.00294 (18)
W3	0.0345 (2)	0.0374 (3)	0.0478 (3)	0.00344 (19)	−0.00085 (19)	0.0026 (2)
V3	0.0345 (2)	0.0374 (3)	0.0478 (3)	0.00344 (19)	−0.00085 (19)	0.0026 (2)
W4	0.0409 (3)	0.0558 (3)	0.0327 (2)	−0.0115 (2)	0.00890 (19)	0.0005 (2)
V4	0.0409 (3)	0.0558 (3)	0.0327 (2)	−0.0115 (2)	0.00890 (19)	0.0005 (2)
W5	0.0452 (3)	0.0292 (2)	0.0711 (3)	−0.0081 (2)	0.0181 (2)	−0.0074 (2)
V5	0.0452 (3)	0.0292 (2)	0.0711 (3)	−0.0081 (2)	0.0181 (2)	−0.0074 (2)
W6	0.0643 (3)	0.0444 (3)	0.0366 (2)	−0.0025 (2)	0.0124 (2)	−0.0138 (2)
V6	0.0643 (3)	0.0444 (3)	0.0366 (2)	−0.0025 (2)	0.0124 (2)	−0.0138 (2)
O1E	0.042 (5)	0.056 (5)	0.129 (8)	0.000 (4)	0.046 (5)	−0.017 (5)
O2E	0.047 (5)	0.093 (7)	0.044 (4)	−0.032 (5)	−0.013 (4)	−0.003 (4)
O3E	0.094 (7)	0.044 (5)	0.062 (5)	0.022 (5)	0.021 (5)	0.014 (4)
O4E	0.046 (4)	0.082 (6)	0.042 (4)	−0.008 (4)	0.010 (4)	0.019 (4)
O5E	0.042 (4)	0.029 (4)	0.079 (6)	−0.007 (3)	−0.003 (4)	−0.001 (4)
O6E	0.071 (6)	0.080 (7)	0.055 (5)	−0.003 (5)	0.005 (4)	−0.041 (5)
O1	0.077 (5)	0.044 (4)	0.034 (4)	−0.018 (4)	0.017 (4)	−0.006 (3)
O2	0.079 (6)	0.042 (4)	0.041 (4)	−0.023 (4)	0.021 (4)	−0.011 (3)
O3	0.084 (6)	0.053 (5)	0.062 (5)	−0.022 (5)	−0.038 (5)	0.019 (4)
O4	0.097 (7)	0.047 (5)	0.072 (6)	−0.027 (5)	−0.045 (5)	0.011 (4)
O5	0.077 (6)	0.043 (4)	0.043 (4)	−0.024 (4)	0.021 (4)	−0.004 (3)
O6A	0.024 (9)	0.051 (11)	0.039 (10)	−0.005 (7)	0.003 (7)	0.003 (8)
O6B	0.028 (9)	0.032 (9)	0.028 (9)	0.005 (7)	−0.002 (6)	0.006 (6)
O7	0.029 (4)	0.124 (9)	0.091 (7)	−0.010 (5)	0.010 (4)	−0.070 (6)
O8	0.081 (6)	0.050 (5)	0.066 (5)	−0.024 (5)	−0.039 (5)	0.019 (4)
O9A	0.019 (8)	0.043 (9)	0.031 (8)	0.003 (7)	−0.006 (6)	0.003 (7)
O9B	0.009 (7)	0.063 (11)	0.028 (8)	−0.004 (7)	−0.007 (5)	−0.003 (7)
O10	0.027 (4)	0.137 (9)	0.092 (7)	−0.007 (5)	0.008 (4)	−0.070 (7)
O11A	0.041 (10)	0.039 (11)	0.044 (12)	0.000 (8)	0.009 (8)	−0.012 (8)
O11B	0.044 (11)	0.045 (11)	0.029 (9)	0.006 (8)	0.019 (8)	−0.010 (7)
O12	0.033 (4)	0.112 (8)	0.095 (7)	−0.008 (5)	0.004 (4)	−0.068 (6)
P	0.0258 (15)	0.0167 (14)	0.0257 (15)	−0.0004 (12)	0.0030 (12)	−0.0015 (12)
O1C	0.027 (6)	0.031 (7)	0.022 (6)	0.001 (5)	−0.003 (5)	0.005 (5)
O3C	0.026 (6)	0.030 (7)	0.030 (6)	0.005 (5)	0.010 (5)	−0.004 (5)
O4C	0.020 (6)	0.038 (7)	0.025 (6)	0.000 (5)	0.008 (5)	−0.001 (5)
O2C	0.025 (6)	0.031 (7)	0.022 (6)	−0.009 (5)	−0.010 (5)	−0.006 (5)
O1W	0.109 (10)	0.069 (7)	0.129 (10)	0.024 (7)	0.057 (8)	0.007 (7)
Co	0.0363 (7)	0.0382 (8)	0.0355 (7)	0.0024 (6)	0.0067 (6)	0.0015 (6)
N1	0.036 (5)	0.074 (7)	0.045 (5)	0.006 (5)	0.013 (4)	−0.004 (5)
N2	0.047 (5)	0.048 (6)	0.048 (5)	0.002 (4)	0.014 (4)	0.000 (4)
N3	0.047 (5)	0.034 (5)	0.041 (5)	0.007 (4)	0.002 (4)	0.000 (4)
N4	0.070 (7)	0.051 (6)	0.039 (5)	0.017 (5)	0.009 (5)	−0.001 (4)
N5	0.041 (5)	0.036 (5)	0.035 (4)	−0.004 (4)	0.005 (4)	0.006 (4)
N6	0.035 (4)	0.043 (5)	0.030 (4)	0.006 (4)	0.000 (3)	−0.005 (4)
C1	0.048 (7)	0.091 (11)	0.056 (8)	0.008 (7)	0.017 (6)	0.000 (7)
C2	0.051 (8)	0.139 (17)	0.058 (9)	0.012 (10)	0.007 (7)	−0.007 (10)
C3	0.076 (11)	0.114 (15)	0.067 (10)	0.040 (11)	0.020 (8)	0.025 (10)
C4	0.070 (9)	0.067 (9)	0.048 (7)	0.029 (7)	0.017 (6)	0.013 (6)
C5	0.103 (12)	0.060 (9)	0.069 (9)	0.040 (9)	0.040 (9)	0.019 (7)
C6	0.099 (12)	0.052 (8)	0.078 (10)	0.022 (8)	0.042 (9)	0.000 (7)
C7	0.077 (9)	0.049 (7)	0.064 (8)	0.008 (7)	0.038 (7)	−0.007 (6)
C8	0.082 (10)	0.057 (9)	0.079 (10)	−0.015 (8)	0.037 (8)	−0.028 (8)
C9	0.080 (10)	0.081 (11)	0.053 (8)	−0.001 (8)	0.006 (7)	−0.028 (8)
C10	0.060 (8)	0.069 (9)	0.050 (7)	−0.020 (7)	0.007 (6)	−0.018 (6)
C11	0.054 (7)	0.045 (7)	0.044 (6)	0.008 (5)	0.023 (5)	0.002 (5)
C12	0.049 (7)	0.068 (9)	0.049 (7)	0.010 (6)	0.020 (6)	0.007 (6)
C13	0.053 (7)	0.041 (7)	0.062 (7)	0.007 (5)	0.013 (6)	−0.005 (6)
C14	0.044 (7)	0.064 (8)	0.068 (8)	0.020 (6)	0.005 (6)	−0.017 (7)
C15	0.041 (6)	0.064 (8)	0.066 (8)	0.017 (6)	0.007 (6)	−0.010 (7)
C16	0.040 (6)	0.054 (7)	0.058 (7)	0.018 (6)	0.005 (5)	0.012 (6)
C17	0.038 (6)	0.048 (7)	0.093 (10)	0.011 (6)	0.009 (6)	0.003 (7)
C18	0.040 (7)	0.043 (7)	0.102 (11)	0.006 (6)	−0.006 (7)	0.015 (7)
C19	0.048 (7)	0.044 (7)	0.070 (8)	0.012 (6)	−0.014 (6)	0.004 (6)
C20	0.065 (9)	0.054 (8)	0.068 (9)	0.010 (7)	−0.022 (7)	0.000 (7)
C21	0.097 (12)	0.055 (9)	0.057 (8)	0.024 (8)	−0.012 (8)	−0.012 (7)
C22	0.085 (10)	0.049 (8)	0.055 (7)	0.017 (7)	0.009 (7)	−0.006 (6)
C23	0.044 (6)	0.045 (6)	0.043 (6)	0.011 (5)	−0.005 (5)	0.005 (5)
C24	0.032 (5)	0.040 (6)	0.053 (6)	0.007 (5)	−0.005 (5)	−0.006 (5)
C25	0.068 (8)	0.059 (8)	0.044 (6)	−0.004 (7)	0.014 (6)	0.007 (6)
C26	0.068 (8)	0.057 (8)	0.046 (7)	0.004 (6)	0.005 (6)	0.029 (6)
C27	0.058 (8)	0.049 (7)	0.060 (8)	−0.010 (6)	−0.014 (6)	0.024 (6)
C28	0.044 (6)	0.038 (6)	0.044 (6)	−0.010 (5)	−0.010 (5)	0.001 (5)
C29	0.032 (6)	0.083 (10)	0.068 (8)	0.004 (6)	−0.010 (6)	0.013 (7)
C30	0.030 (6)	0.104 (12)	0.069 (9)	0.016 (7)	−0.001 (6)	−0.006 (8)
C31	0.030 (5)	0.085 (9)	0.047 (6)	−0.006 (6)	0.010 (5)	−0.012 (6)
C32	0.037 (6)	0.116 (12)	0.050 (7)	0.001 (7)	0.015 (6)	−0.005 (7)
C33	0.052 (7)	0.093 (10)	0.036 (6)	−0.010 (7)	0.010 (5)	0.006 (6)
C34	0.044 (6)	0.069 (8)	0.031 (5)	0.001 (6)	0.005 (5)	0.001 (5)
C35	0.032 (5)	0.037 (5)	0.038 (5)	−0.002 (4)	0.002 (4)	0.002 (4)
C36	0.030 (5)	0.035 (5)	0.041 (5)	−0.010 (4)	−0.004 (4)	−0.002 (4)

### Geometric parameters (Å, º)

**Table:**

W1—O1E	1.677 (7)	N2—C11	1.355 (14)
W1—O4	1.880 (8)	N3—C13	1.342 (13)
W1—O2	1.895 (7)	N3—C24	1.350 (13)
W1—O3	1.909 (8)	N4—C22	1.325 (14)
W1—O1	1.912 (7)	N4—C23	1.357 (15)
W1—O4C	2.481 (11)	N5—C25	1.323 (13)
W2—O2E	1.672 (7)	N5—C36	1.352 (12)
W2—O1	1.893 (7)	N6—C34	1.328 (13)
W2—O5	1.917 (7)	N6—C35	1.348 (12)
W2—O6A	1.917 (18)	C1—C2	1.40 (2)
W2—O9A	1.971 (15)	C1—H1	0.9300
W2—O4C	2.526 (12)	C2—C3	1.35 (2)
W3—O3E	1.667 (8)	C2—H2	0.9300
W3—O5	1.873 (7)	C3—C4	1.42 (2)
W3—O2	1.886 (7)	C3—H3	0.9300
W3—O12	1.893 (8)	C4—C12	1.373 (17)
W3—O10	1.896 (8)	C4—C5	1.45 (2)
W3—O1C^i^	2.396 (11)	C5—C6	1.33 (2)
W3—O4C	2.473 (12)	C5—H5	0.9300
W4—O4E	1.676 (7)	C6—C7	1.45 (2)
W4—O3	1.875 (9)	C6—H6	0.9300
W4—O7	1.887 (8)	C7—C11	1.412 (17)
W4—O10^i^	1.897 (9)	C7—C8	1.41 (2)
W4—O8	1.905 (8)	C8—C9	1.35 (2)
W4—O1C	2.451 (12)	C8—H8	0.9300
W5—O5E	1.669 (7)	C9—C10	1.344 (19)
W5—O6A^i^	1.750 (18)	C9—H9	0.9300
W5—O12	1.897 (9)	C10—H10	0.9300
W5—O7^i^	1.908 (8)	C11—C12	1.421 (17)
W5—O11A	2.012 (19)	C13—C14	1.401 (17)
W5—O1C^i^	2.494 (12)	C13—H13	0.9300
W6—O6E	1.673 (7)	C14—C15	1.376 (18)
W6—O4	1.885 (9)	C14—H14	0.9300
W6—O8	1.887 (8)	C15—C16	1.410 (17)
W6—O11A	1.96 (2)	C15—H15	0.9300
W6—O9A^i^	2.085 (14)	C16—C24	1.426 (16)
O6A—W5^i^	1.750 (18)	C16—C17	1.429 (17)
O6B—V5^i^	2.064 (15)	C17—C18	1.342 (18)
O7—V5^i^	1.908 (8)	C17—H17	0.9300
O7—W5^i^	1.908 (8)	C18—C19	1.429 (19)
O9A—W6^i^	2.085 (14)	C18—H18	0.9300
O9B—V6^i^	1.746 (15)	C19—C23	1.397 (17)
O10—V4^i^	1.897 (9)	C19—C20	1.417 (19)
O10—W4^i^	1.897 (9)	C20—C21	1.36 (2)
P—O4C	1.481 (11)	C20—H20	0.9300
P—O4C^i^	1.481 (11)	C21—C22	1.37 (2)
P—O3C	1.530 (11)	C21—H21	0.9300
P—O3C^i^	1.530 (11)	C22—H22	0.9300
P—O1C^i^	1.554 (12)	C23—C24	1.430 (15)
P—O1C	1.554 (12)	C25—C26	1.366 (16)
P—O2C	1.582 (11)	C25—H25	0.9300
P—O2C^i^	1.582 (11)	C26—C27	1.347 (18)
O1C—O4C^i^	1.698 (16)	C26—H26	0.9300
O1C—W3^i^	2.396 (11)	C27—C28	1.399 (16)
O1C—W5^i^	2.494 (12)	C27—H27	0.9300
O4C—O1C^i^	1.698 (16)	C28—C36	1.413 (14)
O2C—V6^i^	2.453 (12)	C28—C29	1.436 (17)
O2C—V5^i^	2.462 (12)	C29—C30	1.351 (18)
O1W—H1W1	0.851 (10)	C29—H29	0.9300
O1W—H2W1	0.851 (10)	C30—C31	1.436 (17)
Co—N3	2.053 (9)	C30—H30	0.9300
Co—N6	2.060 (8)	C31—C32	1.398 (17)
Co—N1	2.066 (10)	C31—C35	1.406 (14)
Co—N4	2.066 (11)	C32—C33	1.346 (17)
Co—N2	2.078 (10)	C32—H32	0.9300
Co—N5	2.085 (8)	C33—C34	1.398 (16)
N1—C1	1.318 (16)	C33—H33	0.9300
N1—C12	1.369 (16)	C34—H34	0.9300
N2—C10	1.318 (14)	C35—C36	1.432 (14)
			
O1E—W1—O4	102.0 (5)	W3—O4C—W1	91.2 (4)
O1E—W1—O2	101.1 (4)	P—O4C—W2	123.5 (7)
O4—W1—O2	89.3 (4)	O1C^i^—O4C—W2	129.3 (7)
O1E—W1—O3	102.3 (5)	W3—O4C—W2	90.2 (4)
O4—W1—O3	87.7 (3)	W1—O4C—W2	90.8 (4)
O2—W1—O3	156.6 (4)	P—O2C—V6^i^	122.1 (6)
O1E—W1—O1	101.4 (4)	P—O2C—V5^i^	120.4 (6)
O4—W1—O1	156.7 (4)	V6^i^—O2C—V5^i^	91.7 (4)
O2—W1—O1	86.5 (3)	H1W1—O1W—H2W1	116 (2)
O3—W1—O1	87.1 (3)	N3—Co—N6	170.4 (3)
O1E—W1—O4C	159.7 (5)	N3—Co—N1	95.4 (3)
O4—W1—O4C	92.4 (5)	N6—Co—N1	93.8 (3)
O2—W1—O4C	64.5 (4)	N3—Co—N4	80.0 (4)
O3—W1—O4C	92.4 (4)	N6—Co—N4	97.0 (4)
O1—W1—O4C	65.1 (4)	N1—Co—N4	94.0 (4)
O2E—W2—O1	102.2 (4)	N3—Co—N2	97.5 (3)
O2E—W2—O5	101.8 (4)	N6—Co—N2	86.3 (3)
O1—W2—O5	87.4 (3)	N1—Co—N2	80.6 (4)
O2E—W2—O6A	112.9 (6)	N4—Co—N2	173.9 (4)
O1—W2—O6A	83.5 (6)	N3—Co—N5	90.9 (3)
O5—W2—O6A	145.3 (6)	N6—Co—N5	80.1 (3)
O2E—W2—O9A	91.4 (5)	N1—Co—N5	171.6 (4)
O1—W2—O9A	166.4 (5)	N4—Co—N5	92.5 (4)
O5—W2—O9A	90.3 (5)	N2—Co—N5	93.1 (4)
O6A—W2—O9A	90.8 (7)	C1—N1—C12	117.9 (12)
O2E—W2—O4C	160.0 (4)	C1—N1—Co	129.0 (10)
O1—W2—O4C	64.3 (3)	C12—N1—Co	112.9 (8)
O5—W2—O4C	64.5 (4)	C10—N2—C11	118.0 (11)
O6A—W2—O4C	81.4 (6)	C10—N2—Co	130.3 (9)
O9A—W2—O4C	102.7 (5)	C11—N2—Co	111.2 (8)
O3E—W3—O5	100.9 (4)	C13—N3—C24	118.0 (10)
O3E—W3—O2	100.0 (4)	C13—N3—Co	128.4 (8)
O5—W3—O2	88.3 (3)	C24—N3—Co	113.4 (7)
O3E—W3—O12	101.7 (5)	C22—N4—C23	117.6 (12)
O5—W3—O12	157.3 (4)	C22—N4—Co	129.5 (10)
O2—W3—O12	87.4 (4)	C23—N4—Co	112.8 (7)
O3E—W3—O10	102.5 (5)	C25—N5—C36	117.2 (9)
O5—W3—O10	88.7 (4)	C25—N5—Co	130.4 (8)
O2—W3—O10	157.5 (5)	C36—N5—Co	112.2 (6)
O12—W3—O10	86.8 (4)	C34—N6—C35	119.1 (9)
O3E—W3—O1C^i^	159.8 (4)	C34—N6—Co	127.5 (7)
O5—W3—O1C^i^	93.8 (4)	C35—N6—Co	113.2 (6)
O2—W3—O1C^i^	94.0 (4)	N1—C1—C2	123.2 (15)
O12—W3—O1C^i^	64.3 (4)	N1—C1—H1	118.4
O10—W3—O1C^i^	63.9 (5)	C2—C1—H1	118.4
O3E—W3—O4C	159.3 (4)	C3—C2—C1	118.6 (15)
O5—W3—O4C	66.2 (4)	C3—C2—H2	120.7
O2—W3—O4C	64.8 (4)	C1—C2—H2	120.7
O12—W3—O4C	91.9 (5)	C2—C3—C4	120.0 (15)
O10—W3—O4C	93.7 (5)	C2—C3—H3	120.0
O1C^i^—W3—O4C	40.8 (4)	C4—C3—H3	120.0
O4E—W4—O3	103.0 (4)	C12—C4—C3	117.4 (14)
O4E—W4—O7	101.6 (5)	C12—C4—C5	118.4 (13)
O3—W4—O7	88.7 (4)	C3—C4—C5	124.1 (14)
O4E—W4—O10^i^	99.9 (5)	C6—C5—C4	121.0 (13)
O3—W4—O10^i^	157.1 (5)	C6—C5—H5	119.5
O7—W4—O10^i^	88.2 (4)	C4—C5—H5	119.5
O4E—W4—O8	101.9 (4)	C5—C6—C7	121.5 (15)
O3—W4—O8	87.0 (4)	C5—C6—H6	119.3
O7—W4—O8	156.5 (4)	C7—C6—H6	119.3
O10^i^—W4—O8	86.8 (4)	C11—C7—C8	117.7 (13)
O4E—W4—O1C	157.0 (4)	C11—C7—C6	117.8 (14)
O3—W4—O1C	95.6 (4)	C8—C7—C6	124.5 (14)
O7—W4—O1C	65.0 (4)	C9—C8—C7	118.6 (13)
O10^i^—W4—O1C	62.7 (4)	C9—C8—H8	120.7
O8—W4—O1C	92.4 (4)	C7—C8—H8	120.7
O5E—W5—O6A^i^	112.5 (6)	C10—C9—C8	120.3 (14)
O5E—W5—O12	100.4 (4)	C10—C9—H9	119.9
O6A^i^—W5—O12	146.1 (6)	C8—C9—H9	119.9
O5E—W5—O7^i^	100.6 (4)	N2—C10—C9	124.4 (14)
O6A^i^—W5—O7^i^	79.6 (7)	N2—C10—H10	117.8
O12—W5—O7^i^	86.8 (4)	C9—C10—H10	117.8
O5E—W5—O11A	94.4 (6)	N2—C11—C7	121.0 (11)
O6A^i^—W5—O11A	89.5 (8)	N2—C11—C12	119.3 (11)
O12—W5—O11A	96.1 (6)	C7—C11—C12	119.7 (12)
O7^i^—W5—O11A	164.0 (7)	N1—C12—C4	122.8 (12)
O5E—W5—O1C^i^	155.8 (4)	N1—C12—C11	115.7 (11)
O6A^i^—W5—O1C^i^	84.0 (6)	C4—C12—C11	121.4 (13)
O12—W5—O1C^i^	62.1 (4)	N3—C13—C14	122.7 (11)
O7^i^—W5—O1C^i^	63.8 (4)	N3—C13—H13	118.6
O11A—W5—O1C^i^	103.7 (6)	C14—C13—H13	118.6
O6E—W6—O4	101.1 (5)	C15—C14—C13	119.1 (12)
O6E—W6—O8	100.9 (5)	C15—C14—H14	120.4
O4—W6—O8	87.8 (4)	C13—C14—H14	120.4
O6E—W6—O11A	93.7 (6)	C14—C15—C16	120.5 (12)
O4—W6—O11A	93.3 (6)	C14—C15—H15	119.8
O8—W6—O11A	164.8 (6)	C16—C15—H15	119.8
O6E—W6—O9A^i^	94.2 (5)	C15—C16—C24	115.9 (11)
O4—W6—O9A^i^	164.3 (5)	C15—C16—C17	125.1 (12)
O8—W6—O9A^i^	92.6 (5)	C24—C16—C17	119.0 (11)
O11A—W6—O9A^i^	82.3 (7)	C18—C17—C16	120.7 (12)
W2—O1—W1	139.3 (4)	C18—C17—H17	119.7
W3—O2—W1	138.9 (5)	C16—C17—H17	119.7
W4—O3—W1	139.2 (6)	C17—C18—C19	122.0 (13)
W1—O4—W6	139.7 (6)	C17—C18—H18	119.0
W3—O5—W2	138.3 (4)	C19—C18—H18	119.0
W5^i^—O6A—W2	149.7 (10)	C23—C19—C20	116.5 (13)
W4—O7—V5^i^	138.9 (5)	C23—C19—C18	118.9 (12)
W4—O7—W5^i^	138.9 (5)	C20—C19—C18	124.5 (13)
V5^i^—O7—W5^i^	0.00 (3)	C21—C20—C19	119.0 (14)
W6—O8—W4	139.3 (5)	C21—C20—H20	120.5
W2—O9A—W6^i^	123.3 (7)	C19—C20—H20	120.5
W3—O10—V4^i^	138.7 (6)	C20—C21—C22	120.2 (14)
W3—O10—W4^i^	138.7 (6)	C20—C21—H21	119.9
V4^i^—O10—W4^i^	0.00 (3)	C22—C21—H21	119.9
W6—O11A—W5	125.4 (9)	N4—C22—C21	123.2 (14)
W3—O12—W5	139.9 (6)	N4—C22—H22	118.4
O4C—P—O4C^i^	180.0 (8)	C21—C22—H22	118.4
O4C—P—O3C	67.4 (6)	N4—C23—C19	123.3 (11)
O4C^i^—P—O3C	112.6 (6)	N4—C23—C24	116.6 (11)
O4C—P—O3C^i^	112.6 (6)	C19—C23—C24	120.1 (11)
O4C^i^—P—O3C^i^	67.4 (6)	N3—C24—C16	123.8 (10)
O3C—P—O3C^i^	180.000 (3)	N3—C24—C23	116.9 (10)
O4C—P—O1C^i^	68.0 (6)	C16—C24—C23	119.3 (11)
O4C^i^—P—O1C^i^	112.0 (6)	N5—C25—C26	124.1 (12)
O3C—P—O1C^i^	108.6 (6)	N5—C25—H25	117.9
O3C^i^—P—O1C^i^	71.4 (6)	C26—C25—H25	117.9
O4C—P—O1C	112.0 (6)	C27—C26—C25	119.2 (11)
O4C^i^—P—O1C	68.0 (6)	C27—C26—H26	120.4
O3C—P—O1C	71.4 (6)	C25—C26—H26	120.4
O3C^i^—P—O1C	108.6 (6)	C26—C27—C28	120.5 (11)
O1C^i^—P—O1C	180.0 (7)	C26—C27—H27	119.8
O4C—P—O2C	69.2 (6)	C28—C27—H27	119.8
O4C^i^—P—O2C	110.8 (6)	C27—C28—C36	116.3 (11)
O3C—P—O2C	107.1 (6)	C27—C28—C29	124.3 (11)
O3C^i^—P—O2C	72.9 (6)	C36—C28—C29	119.4 (10)
O1C^i^—P—O2C	105.5 (6)	C30—C29—C28	119.3 (11)
O1C—P—O2C	74.5 (6)	C30—C29—H29	120.4
O4C—P—O2C^i^	110.8 (6)	C28—C29—H29	120.4
O4C^i^—P—O2C^i^	69.2 (6)	C29—C30—C31	123.6 (11)
O3C—P—O2C^i^	72.9 (6)	C29—C30—H30	118.2
O3C^i^—P—O2C^i^	107.1 (6)	C31—C30—H30	118.2
O1C^i^—P—O2C^i^	74.5 (6)	C32—C31—C35	116.5 (11)
O1C—P—O2C^i^	105.5 (6)	C32—C31—C30	126.1 (11)
O2C—P—O2C^i^	180.0 (7)	C35—C31—C30	117.4 (11)
P—O1C—O4C^i^	54.0 (6)	C33—C32—C31	120.8 (11)
P—O1C—W3^i^	126.0 (6)	C33—C32—H32	119.6
O4C^i^—O1C—W3^i^	72.0 (6)	C31—C32—H32	119.6
P—O1C—W4	122.9 (7)	C32—C33—C34	119.5 (11)
O4C^i^—O1C—W4	135.7 (7)	C32—C33—H33	120.2
W3^i^—O1C—W4	94.1 (4)	C34—C33—H33	120.2
P—O1C—W5^i^	120.0 (6)	N6—C34—C33	121.5 (11)
O4C^i^—O1C—W5^i^	129.8 (7)	N6—C34—H34	119.2
W3^i^—O1C—W5^i^	93.4 (4)	C33—C34—H34	119.2
W4—O1C—W5^i^	91.9 (4)	N6—C35—C31	122.5 (9)
P—O4C—O1C^i^	58.0 (5)	N6—C35—C36	117.2 (9)
P—O4C—W3	125.2 (6)	C31—C35—C36	120.3 (9)
O1C^i^—O4C—W3	67.2 (5)	N5—C36—C28	122.7 (9)
P—O4C—W1	125.4 (6)	N5—C36—C35	117.3 (9)
O1C^i^—O4C—W1	132.1 (7)	C28—C36—C35	119.9 (9)
			
N3—Co—N1—C1	83.6 (10)	Co—N1—C12—C4	−173.8 (9)
N6—Co—N1—C1	−94.0 (10)	C1—N1—C12—C11	179.9 (10)
N4—Co—N1—C1	3.3 (10)	Co—N1—C12—C11	4.1 (12)
N2—Co—N1—C1	−179.7 (10)	C3—C4—C12—N1	−1.5 (18)
N5—Co—N1—C1	−138 (2)	C5—C4—C12—N1	−178.5 (11)
N3—Co—N1—C12	−101.2 (7)	C3—C4—C12—C11	−179.3 (11)
N6—Co—N1—C12	81.2 (7)	C5—C4—C12—C11	3.8 (17)
N4—Co—N1—C12	178.5 (7)	N2—C11—C12—N1	−0.5 (15)
N2—Co—N1—C12	−4.5 (7)	C7—C11—C12—N1	−177.9 (10)
N5—Co—N1—C12	37 (3)	N2—C11—C12—C4	177.4 (10)
N3—Co—N2—C10	−89.4 (10)	C7—C11—C12—C4	0.0 (16)
N6—Co—N2—C10	81.8 (10)	C24—N3—C13—C14	−1.0 (16)
N1—Co—N2—C10	176.3 (10)	Co—N3—C13—C14	172.4 (9)
N4—Co—N2—C10	−155 (3)	N3—C13—C14—C15	2.1 (18)
N5—Co—N2—C10	1.9 (10)	C13—C14—C15—C16	−1.4 (18)
N3—Co—N2—C11	98.4 (7)	C14—C15—C16—C24	−0.2 (16)
N6—Co—N2—C11	−90.4 (7)	C14—C15—C16—C17	−178.5 (11)
N1—Co—N2—C11	4.1 (7)	C15—C16—C17—C18	177.9 (11)
N4—Co—N2—C11	33 (4)	C24—C16—C17—C18	−0.3 (17)
N5—Co—N2—C11	−170.3 (7)	C16—C17—C18—C19	−0.1 (19)
N6—Co—N3—C13	−105 (2)	C17—C18—C19—C23	−0.3 (18)
N1—Co—N3—C13	88.7 (10)	C17—C18—C19—C20	−178.3 (12)
N4—Co—N3—C13	−178.2 (10)	C23—C19—C20—C21	3.5 (17)
N2—Co—N3—C13	7.5 (10)	C18—C19—C20—C21	−178.4 (12)
N5—Co—N3—C13	−85.8 (9)	C19—C20—C21—C22	−4.4 (19)
N6—Co—N3—C24	68 (2)	C23—N4—C22—C21	−1.6 (18)
N1—Co—N3—C24	−97.7 (7)	Co—N4—C22—C21	−176.6 (10)
N4—Co—N3—C24	−4.5 (7)	C20—C21—C22—N4	4 (2)
N2—Co—N3—C24	−178.8 (7)	C22—N4—C23—C19	0.8 (16)
N5—Co—N3—C24	87.9 (7)	Co—N4—C23—C19	176.6 (8)
N3—Co—N4—C22	−180.0 (11)	C22—N4—C23—C24	179.7 (10)
N6—Co—N4—C22	9.2 (11)	Co—N4—C23—C24	−4.5 (12)
N1—Co—N4—C22	−85.2 (10)	C20—C19—C23—N4	−1.8 (16)
N2—Co—N4—C22	−113 (3)	C18—C19—C23—N4	−179.9 (10)
N5—Co—N4—C22	89.5 (10)	C20—C19—C23—C24	179.3 (10)
N3—Co—N4—C23	4.8 (7)	C18—C19—C23—C24	1.1 (16)
N6—Co—N4—C23	−166.0 (7)	C13—N3—C24—C16	−0.8 (15)
N1—Co—N4—C23	99.7 (8)	Co—N3—C24—C16	−175.2 (8)
N2—Co—N4—C23	71 (4)	C13—N3—C24—C23	177.9 (9)
N5—Co—N4—C23	−85.6 (8)	Co—N3—C24—C23	3.5 (11)
N3—Co—N5—C25	6.6 (10)	C15—C16—C24—N3	1.4 (16)
N6—Co—N5—C25	−176.6 (10)	C17—C16—C24—N3	179.8 (10)
N1—Co—N5—C25	−132 (2)	C15—C16—C24—C23	−177.3 (10)
N4—Co—N5—C25	86.7 (10)	C17—C16—C24—C23	1.1 (15)
N2—Co—N5—C25	−90.9 (10)	N4—C23—C24—N3	0.7 (14)
N3—Co—N5—C36	−179.0 (7)	C19—C23—C24—N3	179.7 (9)
N6—Co—N5—C36	−2.3 (7)	N4—C23—C24—C16	179.5 (9)
N1—Co—N5—C36	42 (3)	C19—C23—C24—C16	−1.5 (15)
N4—Co—N5—C36	−98.9 (7)	C36—N5—C25—C26	0.2 (17)
N2—Co—N5—C36	83.5 (7)	Co—N5—C25—C26	174.4 (9)
N3—Co—N6—C34	−162.2 (18)	N5—C25—C26—C27	1 (2)
N1—Co—N6—C34	3.7 (10)	C25—C26—C27—C28	−1.6 (19)
N4—Co—N6—C34	−90.8 (9)	C26—C27—C28—C36	0.9 (17)
N2—Co—N6—C34	84.0 (10)	C26—C27—C28—C29	−176.9 (13)
N5—Co—N6—C34	177.8 (10)	C27—C28—C29—C30	177.4 (13)
N3—Co—N6—C35	23 (2)	C36—C28—C29—C30	−0.4 (19)
N1—Co—N6—C35	−171.4 (7)	C28—C29—C30—C31	0 (2)
N4—Co—N6—C35	94.0 (7)	C29—C30—C31—C32	−179.1 (15)
N2—Co—N6—C35	−91.2 (7)	C29—C30—C31—C35	0 (2)
N5—Co—N6—C35	2.7 (7)	C35—C31—C32—C33	0 (2)
C12—N1—C1—C2	−1.2 (17)	C30—C31—C32—C33	179.1 (14)
Co—N1—C1—C2	173.8 (9)	C31—C32—C33—C34	−1 (2)
N1—C1—C2—C3	0 (2)	C35—N6—C34—C33	−2.3 (17)
C1—C2—C3—C4	0 (2)	Co—N6—C34—C33	−177.2 (9)
C2—C3—C4—C12	0 (2)	C32—C33—C34—N6	2 (2)
C2—C3—C4—C5	177.0 (14)	C34—N6—C35—C31	0.8 (16)
C12—C4—C5—C6	−5.0 (19)	Co—N6—C35—C31	176.4 (9)
C3—C4—C5—C6	178.3 (13)	C34—N6—C35—C36	−178.3 (9)
C4—C5—C6—C7	2 (2)	Co—N6—C35—C36	−2.7 (11)
C5—C6—C7—C11	1.5 (18)	C32—C31—C35—N6	0.5 (17)
C5—C6—C7—C8	−179.5 (13)	C30—C31—C35—N6	−178.9 (11)
C11—C7—C8—C9	−2.1 (18)	C32—C31—C35—C36	179.6 (11)
C6—C7—C8—C9	178.9 (12)	C30—C31—C35—C36	0.1 (17)
C7—C8—C9—C10	2 (2)	C25—N5—C36—C28	−1.0 (15)
C11—N2—C10—C9	−1.5 (18)	Co—N5—C36—C28	−176.1 (8)
Co—N2—C10—C9	−173.2 (10)	C25—N5—C36—C35	176.7 (10)
C8—C9—C10—N2	0 (2)	Co—N5—C36—C35	1.5 (11)
C10—N2—C11—C7	0.8 (15)	C27—C28—C36—N5	0.4 (15)
Co—N2—C11—C7	174.1 (8)	C29—C28—C36—N5	178.4 (10)
C10—N2—C11—C12	−176.5 (10)	C27—C28—C36—C35	−177.2 (10)
Co—N2—C11—C12	−3.3 (12)	C29—C28—C36—C35	0.7 (15)
C8—C7—C11—N2	0.9 (16)	N6—C35—C36—N5	0.8 (13)
C6—C7—C11—N2	180.0 (10)	C31—C35—C36—N5	−178.4 (10)
C8—C7—C11—C12	178.3 (10)	N6—C35—C36—C28	178.5 (9)
C6—C7—C11—C12	−2.7 (16)	C31—C35—C36—C28	−0.6 (15)
C1—N1—C12—C4	2.0 (16)		

Symmetry code: (i) −*x*+3/2, −*y*+1/2, −*z*+1.

### Hydrogen-bond geometry (Å, º)

Cg1 and Cg2 are the centroids of the N1/C1–C4/C12 and N3/C13–C16/C24 rings, respectively.

**Table:**

*D*—H···*A*	*D*—H	H···*A*	*D*···*A*	*D*—H···*A*
O1*W*—H1*W*1···O1*E*	0.85 (1)	2.17 (11)	2.928 (14)	149 (20)
O1*W*—H2*W*1···O3*E*^ii^	0.85 (1)	1.99 (3)	2.836 (13)	173 (17)
C9—H9···O5*E*^iii^	0.93	2.55	3.208 (15)	128
C26—H26···O12^iv^	0.93	2.46	2.973 (15)	114
C33—H33···O5^i^	0.93	2.53	3.345 (13)	147
C34—H34···O8	0.93	2.43	3.114 (13)	130
C34—H34···*Cg*1	0.93	3.04	3.811 (12)	142
C25—H25···*Cg*2	0.93	2.99	3.777 (13)	143

Symmetry codes: (i) −*x*+3/2, −*y*+1/2, −*z*+1; (ii) −*x*+2, −*y*, −*z*+1; (iii) *x*, −*y*, *z*−1/2; (iv) −*x*+3/2, *y*+1/2, −*z*+1/2.
